# A new stochastic diffusion model for influence maximization in social networks

**DOI:** 10.1038/s41598-023-33010-8

**Published:** 2023-04-14

**Authors:** Alireza Rezvanian, S. Mehdi Vahidipour, Mohammad Reza Meybodi

**Affiliations:** 1 Department of Computer Engineering, University of Science and Culture, Tehran, Iran; 2 Computer Engineering Department, Faculty of Electrical and Computer Engineering, University of Kashan, Kashan, Iran; 3 Computer Engineering Department, Amirkabir University of Technology, (Tehran Polytechnic), Tehran, Iran

**Keywords:** Physics, Information theory and computation

## Abstract

Most current studies on information diffusion in online social networks focus on the deterministic aspects of social networks. However, the behavioral parameters of online social networks are uncertain, unpredictable, and time-varying. Thus, deterministic graphs for modeling information diffusion in online social networks are too restrictive to solve most real network problems, such as influence maximization. Recently, stochastic graphs have been proposed as a graph model for social network applications where the weights associated with links in the stochastic graph are random variables. In this paper, we first propose a diffusion model based on a stochastic graph, in which influence probabilities associated with its links are unknown random variables. Then we develop an approach using the set of learning automata residing in the proposed diffusion model to estimate the influence probabilities by sampling from the links of the stochastic graph. Numerical simulations conducted on real and artificial stochastic networks demonstrate the effectiveness of the proposed stochastic diffusion model for influence maximization.

## Introduction

In recent years, analysis of social networks, particularly information diffusion through online social networks (OSNs), has received increasing attention^1–4^. OSNs such as *Twitter* and *Facebook* have facilitated several online services to enable users to engage in friendship activities, facilitate interactions between users, and to enable users to broadcast things that happen in their daily lives, such as what they are reading, thinking, and experiencing, or to post information about the latest news and share user-generated content from other users through social networks. Information, news, events, ideas, innovations, behaviors, and trends can spread via taking interactions between online social users, and information content generated by influential users can cascade to friends of friends over the online social network via online human activities such as sharing, reposting, liking or commenting on the given information content. Accordingly, a small number of influential initial users can yield widespread diffusion of information by the so-called *word-of-mouth* effect. For this purpose, one may analyze the structural parameters of the network using network centrality measures^5^ and identify the seed set of influential users using node degree centrality measure; this leads to finding users with the maximum number of friends. However, this may not lead to finding the best seed set of influential users because the importance of influential users as influential leaders of the network may not necessarily consist of having many friends.

In addition, for the process of influencing users to adopt and spread a particular piece of information or behavior, the influence probability between pairs of users may be more important than the number of their friends because it seems reasonable that users with higher influence probabilities can influence users with lower influence probabilities to adopt a particular behavior or propagate certain kind of activities for achieving marketing goals. However, most studies only consider the deterministic effect of spreading on a deterministic graph with fixed influence probabilities. Thus, they cannot be adopted for characterizing OSNs, where user interactions behave differently over time. It does not seem reasonable to expect deterministic activities or user interactions without changes over time for each user. Furthermore, in diffusion models with fixed influence probabilities, it is assumed that the network information users are available and remain unchanged during the lifetime of the network. In practice, the influence probabilities of users are not known; users’ activities such as their friendship behavior, frequency of receiving comment(s)/like(s) on a wall, and sharing of tweets change over time, can be used to estimate the influence probabilities^6^.

However, if stochastic graphs are chosen for graph modeling of an OSN^6^, the concept of stochastic graph centrality measures and the diffusion models for stochastic graphs in which influence probabilities are random variables; are more suitable for studying the network.

Accordingly, due to the unpredictable, uncertain, and time-varying nature of OSNs^7^, deterministic approaches to users' social activities and their influence probabilities reflect only a snapshot of a network and ignore the continuum of user activities and interactions occurring over time. Thus, it seems that the stochastic graph model is a better alternative as a new graph model for real network problems with a time-varying nature^6^. Link weights in stochastic graphs are random variables. Once the graph model of an OSN is chosen to be a stochastic graph, the spread of influence under given diffusion models and the influence probabilities associated with the interactions of online users as random variables could be redesigned to consider the stochastic nature of the model. Several applications of modeling stochastic graphs for OSNs have been reported in the literature^6,8–12^.

This paper based on the above justification and our previous study on network centralities utilizes the stochastic graph to model the time-varying nature network. Thus, this paper, proposes a diffusion model for social networks using a stochastic graph whose weights associated with the links represent activities between two nodes in the stochastic graph. In the proposed model, the influence probability (IP), defined for a link, indicates the probability that a connected node is influenced by another node at the end of the link. The defined influence probabilities in the model are computed using the weights of links. Since these weights are random variables with unknown distribution functions (PDF), the computed influence probabilities are also positive random variables with unknown PDFs. To estimate the distribution of influence probabilities, we propose a learning automata-based algorithm, where the estimation is computed by taking samples from the weight links, which are random variables with an unknown PDF.

In summary, the main contributions of this paper are:

- Utilizing the stochastic graph model to consider the time-varying nature of the influence probability of the information diffusion model.
- Proposing a diffusion model as the stochastic graph for influence maximization.
- Designing an algorithm for estimation of influence probabilities on the stochastic model of the diffusion model.

A few studies tried to model user behavior prediction^13–15^ however they did not aim to influence maximization. Most current studies on information diffusion in online social networks focus on the deterministic aspects of social networks. It means that they consider a graph with fixed weights for parameters. However, the behavioral parameters of online social networks are uncertain, unpredictable, and time-varying. Thus, deterministic graphs for modeling information diffusion in online social networks are too restrictive to solve most real network problems, such as influence maximization. Recently, stochastic graphs have been proposed as a graph model for social network applications where the weights associated with links in the stochastic graph are random variables. In this paper, we first propose a diffusion model based on a stochastic graph, in which influence probabilities associated with its links are unknown random variables. Then we develop an approach using the set of learning automata residing in the proposed diffusion model to estimate the influence probabilities by sampling from the links of the stochastic graph. Numerical simulations conducted on real and artificial stochastic networks demonstrate the effectiveness of the proposed stochastic diffusion model for influence maximization.

## Related work

The first study on influence maximization was done by *Domingos* et al.^16^, who represented a market as a social network and modeled the influence between users as a Markov random field. Then, in a seminal study, *Kempe* et al.^17^ presented a discrete optimization problem for influence maximization. These authors showed that the maximization problem is NP-hard in the independent cascade model (ICM)^18^ and the linear threshold model (LTM)^19^. They also presented a greedy algorithm with a worst-case guarantee of being within (1–1/*e*) of the optimal solution for maximizing the spread of influence (where *e* is the base of the natural logarithm). However, heavy Monte Carlo simulations are required in this approach to estimate the influence spreads of different seed sets. Thus, many advanced greedy algorithms^20–22^ have been proposed to speed up seed-set selection. In^23^, *Kimura* et al. provided an influence cascade model based on the shortest path and an efficient algorithm to calculate the information spread under this model. In^20^, *Leskovec* et al. introduced a “lazy-forward” optimization method for choosing the seed nodes. They showed experimentally that their method runs 700 times faster than the standard greedy algorithm introduced in^17^. In^24^, *Chen* et al. proposed a heuristic method, called maximum influence arborescence, for the general ICM. However, these algorithms heuristically are only considered the particular features of the ICM. In^25^, the authors presented their degree discount heuristic algorithm for ICM. These methods focused on pruning unnecessary *Monte Carlo* simulations when selecting new influential nodes. In^26^, the authors adopted different models and considered the spreading of influence in viral marketing to estimate the final fraction of buyers. The notions of extracting community structures and identifying the most influential nodes were also investigated by researchers in recent years such as a recent study by Kumar et al.^27^. In this study, the authors considered bridges nodes and communities and presented a Communities-based Spreader Ranking algorithm. This method is based on the structures of networks consisting of community density, community modularity, and community diversity. Besides the model is binary and deterministic. In^28^ the idea and successful attempts of influence maximization studies are applied to biological signaling and regulatory networks as Boolean networks. In^29^ an optimal pruning algorithm with an adjustment ranking is proposed for influence maximization to cope with the time complexity and optimality of the solution. The model is according to the deterministic and binary graph and the main idea for pruning is based on the minimum dominating sets. Li et al.^30^ assumed that each node is associated with a topic and this topic is impacted by the spread of influences between users. The authors concentrated on the definition of the new problem as topic-aware influence maximization and also they presented a heuristic algorithm for solving it.

Other directions of research have been focused on design issues in assigning link weights as the influence probabilities of diffusion models, such as (1) assigning a fixed small weight for all influence probabilities of graph^31^, (2) assigning a fixed weight randomly among a set of weights or drawing from distribution for all influence probabilities of the graph^32^, (3) assigning an equal probability to all incoming links of a node^31,33^ and (4) assigning influence probabilities based on the activities of users as an estimation or learning the influences^34^. The assumptions for these methods, where the same treatment is used for all or many nodes, are unrealistic because the number of interactions and the influences between users may not be the same in social relations. Therefore, in recent studies, researchers have tried to present methods of assigning influence probabilities from the activities of users.

Most of the studies mentioned provide insights into understanding the information diffusion dynamics in OSNs^35^; however, some researchers tried to deal with the uncertainty of the model and data as a robust optimization approach. For example^36^, *He *et al*.* defined a framework for robust influence maximization and then designed an algorithm to discover seed nodes from different models and parameters. In addition, *Kermani *et al. focused on the probabilistic nature of the influence maximization problem and presented an algorithm based on a scenario-based robust optimization method to identify the seed nodes^37^. Both methods are brilliant studies, however in this paper; we consider the time-varying nature of the network by modeling the network as a stochastic graph model. It means that the network parameters can change continuously similar to real scenarios.

Further, most diffusion models only focus on the topological structure of users in OSN^22,25,38^. However, the social relationships between users and the behavior of user interactions can change with time because an activity performed by users or a behavior adopted by users may be influenced by the type and amount of behavior exhibited by their friends, acquaintances, or neighbors, and each user behaves differently over time. Hence, deterministic approaches cannot be adopted to characterize online social networks for information diffusion. They ignore the time-varying nature of user behaviors and user interactions in the social network. Thus, deterministic models can not reflect the time-varying nature of networks for these objectives.

### Learning automata theory

**Figure 1:**
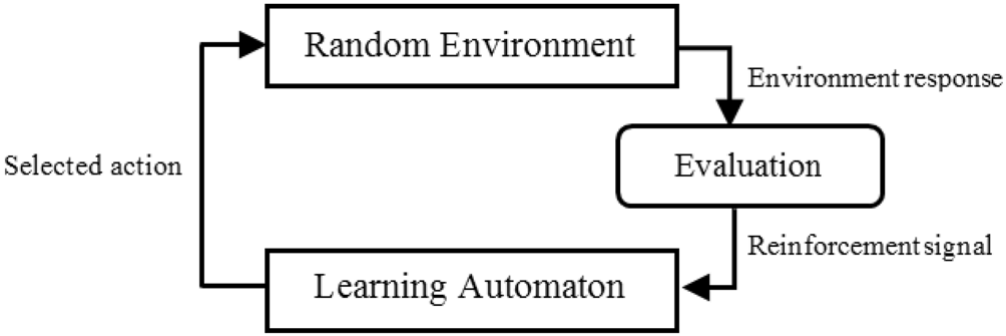
Relationship between LA and its random environment.

A learning automaton (LA)^39^ is an adaptive decision-making model which enhances its performance by learning how to select the optimal action among a limited set of actions from repeated interaction with a random environment. At each time, LA randomly selects an action from its set of possible actions according to its action probability distribution, retained over the set of actions. The selected action is then performed in a random environment. The environment evaluates the selected action and generates a reinforcement signal for LA. LA updates its action probability vector according to the selected action and the received reinforcement signal. Ultimately, LA approaches an action (*i.e.*, the optimal action) to achieve the minimum average penalties from the environment. Figure 1 shows the relationship between an LA and its random environment.

A variable structure LA (VSLA) is described by a 4-tuple $\{\alpha , \beta , p, T\}$, where $\alpha = \{{\alpha }_{1}, \dots , {\alpha }_{r}\}$ denotes the action-set of the LA, *β* = {*β*_1_, …, *β*_*m*_} is the set of inputs, $p = \{{p}_{1}, \dots , {p}_{r}\}$ defines the set of probabilities of actions, and *T* is the learning scheme (*a.k.a.*, the learning algorithm). The learning scheme refers to a recurrence equation, as in $p(t+1)=T[\alpha (t), \beta (t), p(t)]$ for updating the probability vector of actions. A variable structure LA works as follows. The LA selects an action, let the selected action be ${\alpha }_{i}(k)$ using $p(t)$. ${\alpha }_{i}(k)$ applied to the environment, and reinforcement feedback $\beta (t)$ is received from the environment. The LA updates $p(t)$ according to Eq. (1) if $\beta \left(t\right)=0$ (*i.e.,* ${\alpha }_{i}(t)$ is rewarded by the random environment); The LA updates $p(t)$ according to Eq. (2) if $\beta \left(t\right)=1$ (*i.e.*, ${\alpha }_{i}(t)$ is penalized by the random environment).

1 $$p_{j} \left( {t + 1} \right) = \left\{ {\begin{array}{*{20}l} {p_{j} \left( t \right) + a\left( t \right).\left[ {1 - p_{j} \left( t \right)} \right]} \hfill \\ {\left( {1 - a\left( t \right)} \right).p_{j} \left( t \right)} \hfill \\ \end{array} \begin{array}{*{20}c} {j = i} \\ {\forall j \ne i} \\ \end{array} } \right.$$

2 $$p_{j} \left( {t + 1} \right) = \left\{ {\begin{array}{*{20}l} {\left( {1 - b\left( t \right)} \right).p_{j} \left( t \right)j = i} \hfill \\ {\left( {\frac{{b\left( t \right)}}{{r - 1}}} \right) + \left( {1 - b\left( t \right)} \right).p_{j} \left( t \right)\forall j \ne i} \hfill \\ \end{array} } \right.$$

In Eqs. (1) and (2), ${\alpha }_{i}(t)\in \alpha (t)$ is the selected action and $p(t)$ is the probability vector of actions, respectively at time-step $t$. In addition, $a(t) \ge 0$ is a reward parameter, $b(t) \ge 0$ is a penalty parameter and $r$ is the number of actions that can be applied by LA at time-step $t$.

In recent years, LAs have been successfully applied as optimization tools in various applications with unknown, dynamic, and complex environments, such as image processing^40^, optimization^41^, clustering^42^, community detection^43^, cellular networks^44^, wireless sensor networks^45^, queuing systems^46^, grid systems^47^, cloud computing^48^ and complex social networks^49^.

### Stochastic graphs

A stochastic graph $G$ can be defined by a triple $G = \langle V, E, W \rangle$, where $V=\{{v}_{1}, \dots , {v}_{n}\}$ is the set of nodes, $E=\{{e}_{ij}\}\subseteq V\times V$ is the set of links, and $W=\{{w}_{ij} | {e}_{ij}\in E\}$ is the set of weights associated with the links of the graph, in which ${w}_{ij}$ is the random variable associated with link ${e}_{ij}$ between node ${v}_{i}$ and node ${v}_{j}$, if such a link ${e}_{ij}$ exists.

Choosing a stochastic graph as a graph model implies that every concept and algorithm of the network should be treated stochastically. For example, in^6^, some network measures and centralities such as the clustering coefficient, shortest path, degree, strength, betweenness, and closeness, applicable to stochastic graphs, are defined, and some learning automaton-based algorithms for computing them have been designed.

## Diffusion model in stochastic graphs

In this section, we describe the proposed diffusion model, in which a stochastic graph models the spread of influence in OSN. We assume that the probability of influence between two users is an unknown random variable, and our proposed model must compute an estimate for this probability. To this end, we propose an algorithm for the proposed model using learning automata. The proposed diffusion model of the social network is defined by a triple $\mathcal{A}=\langle G, A, IP \rangle$, where:

- $G=\langle V, E, W \rangle$ is a stochastic graph. $V=\{{v}_{1}, {v}_{2},\dots , {v}_{n}\}$ denotes the node-set representing users with $n=|V|$ nodes, $E=\{{e}_{ij}\}\subseteq V\times V$ is the link-set representing a type of relationship between pairs of users with $m=|E|$ links and $W=\{{w}_{ij}\}$ denotes the set of random variables, each of which is associated with a link to the network and which can model any activity between pairs of users. It is assumed that ${w}_{ij}$ is a positive random variable with an unknown PDF.
- $A=\left\{{A}_{ij}\right|{e}_{ij}\in E\}$ is a set of LAs such that ${A}_{ij}$ is assigned to ${e}_{ij}$. This LA tries to estimate the distributions of link weights; ${\widehat{w}}_{ij}\left(t\right)$ indicates the estimation of ${w}_{ij}$ up to time step $t$. The operation of the LAs in the proposed diffusion model will be described later.
- $IP=\left\{{IP}_{ij}\right|{e}_{ij}\in E\}$ represents a matrix in which each element is a random variable and ${IP}_{ij}(t)$ is the probability that node ${v}_{j}$ can be influenced by node ${v}_{i}$ until time step $t$ in $\mathcal{A}$. Further details on $IPs$ are given in the following paragraph.

Consider node ${v}_{i}$ (or the user associated with this node) in the proposed diffusion model $\mathcal{A}$ at time step $t$. This user can influence or adopt a particular behavior at the current time step. In other words, the node (user) ${v}_{i}$ can be active or inactive at a given time step. Since we follow the ICM, the diffusion process evolves in discrete steps, and at time step $t$, every inactive node can become active but active nodes cannot become inactive nodes. Furthermore, in ICM, when a user becomes active, it has an independent chance to make each of its neighboring users active, using the corresponding influence probability. Thus, in the proposed diffusion model, we define a random variable for each link between node ${v}_{i}$ and node ${v}_{j}$_*,*_ referred to as influence probability $I{P}_{ij}\in [0, 1].$ $I{P}_{ij}(t)$ indicates the probability that node ${v}_{j}$ can be influenced by node ${v}_{i}$ at time step $t$, provided that node ${v}_{i}$ has been activated at time step $t-1$. The influence probability of a link from node ${v}_{i}$ to node ${v}_{j}$ can be computed as follows

3 $$IP_{ij} \left( t \right) = \frac{{\hat{w}_{ij} \left( t \right)}}{{\mathop \sum \nolimits_{{v_{k} \in N\left( {v_{j} } \right)}} \hat{w}_{kj} \left( t \right)}}$$

where $N({v}_{j})$ denotes the set of neighboring nodes of ${v}_{j}$ and ${\widehat{w}}_{ij}\left(t\right)=\frac{{w}_{ij\left(1\right)+{w}_{ij}\left(2\right)+\dots {w}_{ij}(t-1)}}{t-1}$ denotes an estimation of the weight of link ${e}_{ij}$ , i.e., ${w}_{ij}$, at time step $t$. The random variable ${w}_{ij}$ represents the number of activities taken from user ${v}_{i}$ to user ${v}_{j}$ that can have been effected on user ${v}_{j}$. The denominator of the above equation is a random variable, which indicates the number of all activities received from all neighbors of node ${v}_{j}$. This is because the process of changing from an inactive node to an active node is based on the probability that the node is affected by its neighboring nodes.

In the following sub-section, we describe the proposed algorithm for computing an estimate of influence probabilities.

### Estimation of influence probabilities

In this subsection, we propose an LA-based algorithm to compute an estimation of the influence probabilities of the stochastic graph in a situation where the PDFs of the influence probabilities associated with the links of the input graph are unknown. The proposed algorithm takes samples from the links of the stochastic graph and then tries to estimate the distribution of the influence probabilities. The process of sampling from the links of the graph is guided with the aid of a set of LA in such a way that 1) the number of samples needed from the links of the stochastic graph for estimating the influence probabilities is as low as possible, and 2) more samples are obtained from those links that reflect a higher rate of change, rather than taking unnecessary samples equally from all links of the graph.

The proposed algorithm consists of initialization, updating, and termination steps. The details of these steps are given below.

#### Initialization

Consider the proposed diffusion model $\mathcal{A}=\langle G, A,IP \rangle$. According to our assumption, the weights of links are positive random variables with unknown PDFs. The proposed algorithm uses the set of LAs in the model, where learning automaton ${A}_{ij}\in A$ is assigned to link ${e}_{ij}\in E$. ${A}_{ij}$ has two actions, since ${\alpha }_{ij}=\{{\alpha }^{1},{\alpha }^{2}\}$, where action ${\alpha }^{1}$ is “take a sample from the link ${e}_{ij}$” and action ${\alpha }^{2}$ is “do not take a sample from the link ${e}_{ij}$”. By sampling from the link ${e}_{ij}$, ${A}_{ij}$ updates its estimate of the weight of ${e}_{ij}$ at the current time step. Let the probability of action of ${A}_{ij}$ at time step $t$ be denoted by ${p}_{ij}(t)=\{{p}_{ij}^{1}, {p}_{ij}^{2}\}$, where ${p}_{ij}^{1}$ and ${p}_{ij}^{2}$ are the probabilities of choosing action *α*^1^ and *α*^2^, respectively. Initially ${p}_{ij}^{1}$=${p}_{ij}^{2}$=1/2 for all ${A}_{ij}\in A$. At the initialization step, the initial values of the estimates of link weights are calculated using several random samples. These samples are taken to provide a general estimate of the distribution of link weights and influence probabilities.

#### Updating

In this step, all learning automata perform in parallel; each learning automaton selects an action based on its action probability vector. If an LA selects the action “taking a sample” (*i.e.*, action ${\mathrm{\alpha }}^{1}$), then a sample is taken from the corresponding link. Using this new sample, the new estimates for the unknown distributions of the link weights ${\widehat{w}}_{ij}(t)$ and the influence probabilities $I{P}_{ij}(t)$ are then computed. Based on the quality of similarity between the new estimate for the distribution of link weights ${\widehat{w}}_{ij}(t)$ and the estimate for the distribution of link weights ${\widehat{w}}_{ij}(t-1)$ obtained in the previous iterations, the reinforcement signal ${\beta }_{ij}(t)$ is computed for each ${A}_{ij}$ according to the following equation:

4 $$\beta_{ij} \left( t \right) = \left\{ {\begin{array}{*{20}l} 0 \hfill & { if (D_{KL} (\hat{w}_{ij} \left( {t - 1} \right)||\hat{w}_{ij} \left( t \right)) >{\varepsilon_p} } \hfill \\ 1 \hfill & {otherwise } \hfill \\ \end{array} } \right.$$

where ${\upbeta }_{ij}\left(t\right)=0$ is the reward signal and ${\upbeta }_{ij}\left(t\right)=1$ is the penalty signal for taking a sample from link ${e}_{ij}$ at instant $t$ and ${D}_{KL}$ denotes the *Kullback–Leibler* divergence (abbreviated as $KL$); specifies the difference between two probabilities $F$ and $F\mathrm{\prime}$ as follows:

5 $$D_{KL} \left( {F || F^{\prime}} \right) = \mathop \sum \limits_{i} F\left( i \right)log\left( {\frac{F\left( i \right)}{{F^{\prime}\left( i \right)}}} \right)$$

Based on the ${\beta }_{ij}(t)$ generated using Eq. (4), all LA residing in the links of the graph update the probabilities of actions according to the learning algorithm.

#### Termination

The algorithm has two termination conditions: (1) the average difference between the estimated influence probability distribution for link weights in two consecutive iterations based on the $KL$ divergence becomes lower than a pre-defined threshold ${T}_{min}$ ; (2) the number of iterations $t$ surpasses a pre-specified threshold ${T}_{max}$.

**Figure 2:**
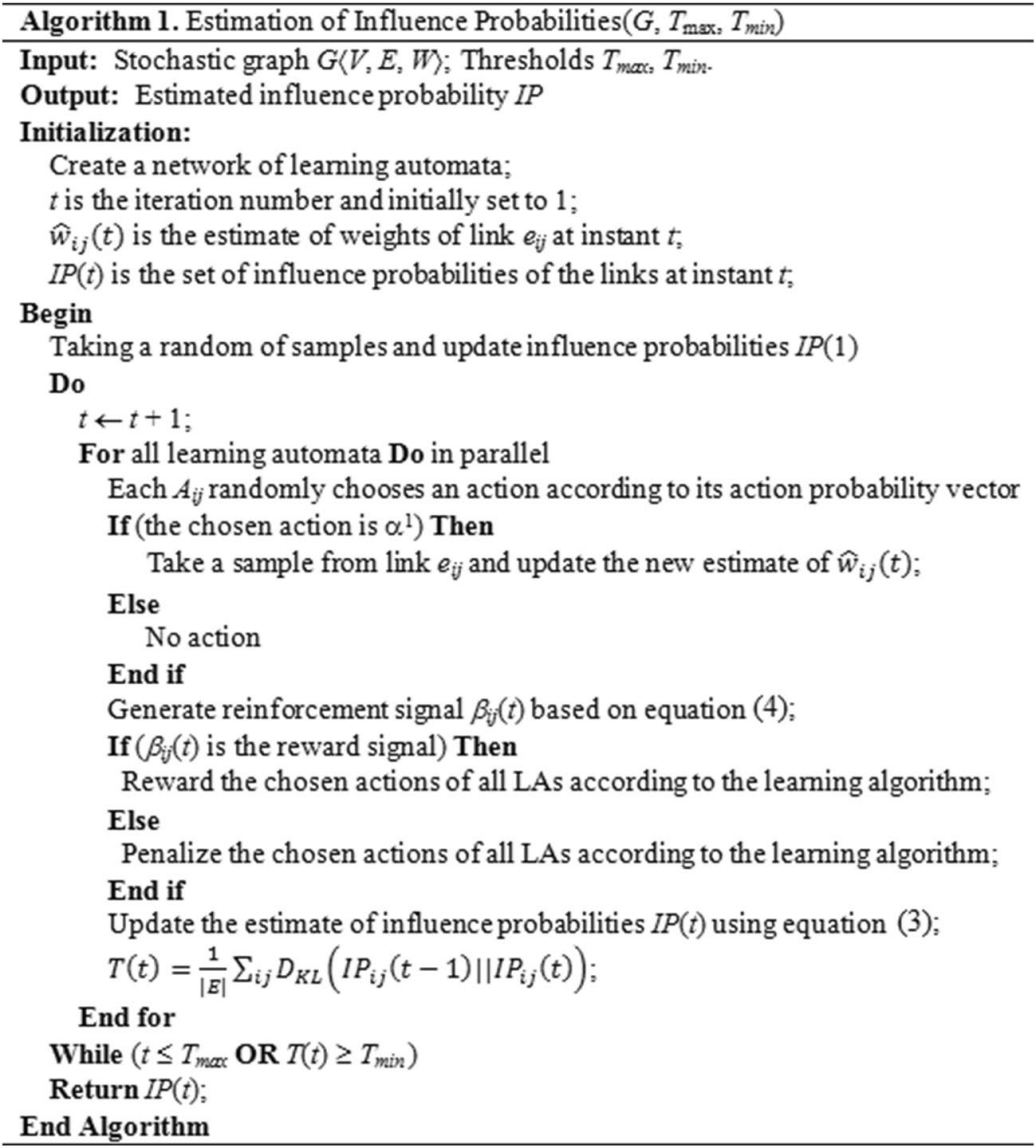
LA-IP algorithm for estimation of influence probabilities (LA-IP).

The pseudo-code of the proposed LA-based algorithm for computing the estimations of distributions of influence probabilities is given in Fig. 2. We call this algorithm *LA-IP*.

### Simulation Experiments

In this section, we conducted several experiments on real and artificial stochastic networks to evaluate the proposed stochastic diffusion model and the proposed algorithms for identifying the influential nodes for influence maximization. In the following subsections, we first describe the test networks and the parameter settings for the experiments and then report the experimental results on the real and artificial stochastic networks.

#### Stochastic test networks

For the experiments, we used several real and artificial stochastic networks. These test networks are described in Table 1. The first set of networks consisted of four real networks: *Ca-cit-HepPh*^50^, *Facebook-wosn-wall*^51^, *Munmun_Digg_reply*^52^, and, *Slashdot-threads*^53^. The second set of networks consisted of two well-known artificial networks, which generate randomly based on well-known computer-generated network models and are commonly used for network analysis. These artificial networks are the *Barabási–Albert* model^54^ (BA-SG), a famous model for generating a scale-free network, and the *Watts–Strogatz* model^55^ (WS-SG), a well-known model for generating a small world network. All these networks are also given in Supplementary file.

**Table 1:** Test Networks for The Experimentation.

Network	Node	Link	Activity	Type
*Ca-cit-HepPh*	28,093	667,331	4,596,803	Undirected author–author collaborations
*Facebook-wosn-wall*	46,952	274,086	8,769,913	Directed user-user wall post
*Munmun_Digg_reply*	30,398	87,627	876,247	Directed user-user reply
*Slashdot-threads*	51,083	131,175	140,778	Directed user-user reply
*BA-SG*	10,000	99,945	749,587	Directed artificial stochastic *Barabási*–*Albert* model
*WS-SG*	10,000	841,633	1,262,449	Directed artificial stochastic *Watts*–*Strogatz* model

All real networks are freely available at http://konect.cc/networks.

#### Experimental setup

In the experiments, the parameter settings for the artificial network models were as follows: number of nodes for all artificial networks $n=10,000$, for the *Barabási*–*Albert* model ${m}_{0}=m=5$ and for the *Watts*–*Strogatz* model $p=0.2$ with $k$ chosen from the integer values in the interval $2 \le k \le 10$. For the experiments using real networks, we have a stream of events for each network, each indicating an activity from a source user to a destination user at a particular time. We used the information about these events to compute the estimates of the distributions of the number of activities occurring on each link in one unit of time, which were subsequently used to estimate the influence probabilities between nodes. Note that a link weight is the distribution of the number of activities occurring between users at both ends of that link, which can be estimated using the stream of events. The link weights of artificial networks are random variables with a *Weibull* distribution, with parameters $a=0.32$ and $b=0.17$. These values have been chosen from an experimental study on *Twitter* for the distribution of lifetime tweets^56^.

For the termination criteria of the LA-IP, the threshold for the maximum number of iterations ${T}_{max}$ is $n\times 100$, where $n$ is the number of nodes for each test network, and the threshold for the minimum average difference between the estimated influence probability distributions for link weights in two consecutive iterations based on the $KL$ divergence ${T}_{min}$, is set to 0.01. The value of ${\varepsilon }_{p}$ for generating a reinforcement signal in the proposed algorithms for learning, influence probabilities are set to 0.05. For LA-IP, the reinforcement learning used for updating the action probability vector is *L*_*R-I,*_ and the learning rate $a$ is set to 0.01. Each experiment was carried out 30 times on a PC with a 3.6 GHz Intel (R) Core i7 CPU and 8 GB of memory. The average results are reported in the following figures and tables.

#### Experimental results

##### Experiment I

This experiment was conducted to evaluate the performance of the LA-based algorithm for estimation of influence probabilities (LA-IP) for predicting activating influences in terms of precision, recall, and F-measure, defined as follows.

*Precision* is computed as the number of corrected activities predicted using estimated influence probabilities by the algorithm, divided by the total number of the items predicted by the algorithm according to the following equation:

6 $$Precision = \frac{\# Correct\;predicted\; items}{{\# Total \;items \;predicted \;by\; the \;algorithm}}$$

*Recall* is proportional to the number of corrected activities predicted using estimated influence activities by the proposed algorithm divided by the total number of activities occurring in reality, as in the following equation:

7 $$Recall = \frac{\# Correct\; predicted \;items}{{\# Total\; known\; items}}$$

*F-measure*, computed as the harmonic mean of the recall and the precision, is a test measure of accuracy that combines these measures. The F-measure is computed according to the following equation

8 $$F - measure = \frac{2\times\;Precision\;\times Recall}{{Precision + Recall}}$$

For this experiment, we consider 80% of the beginning sequence of user activities for learning influence probabilities using the proposed algorithm and the remaining 20% of the last sequence of user activities for testing the algorithms concerning the measures described. The initial seed-set size *k* for this experiment was 50 for all the test networks.

**Figure 3:**
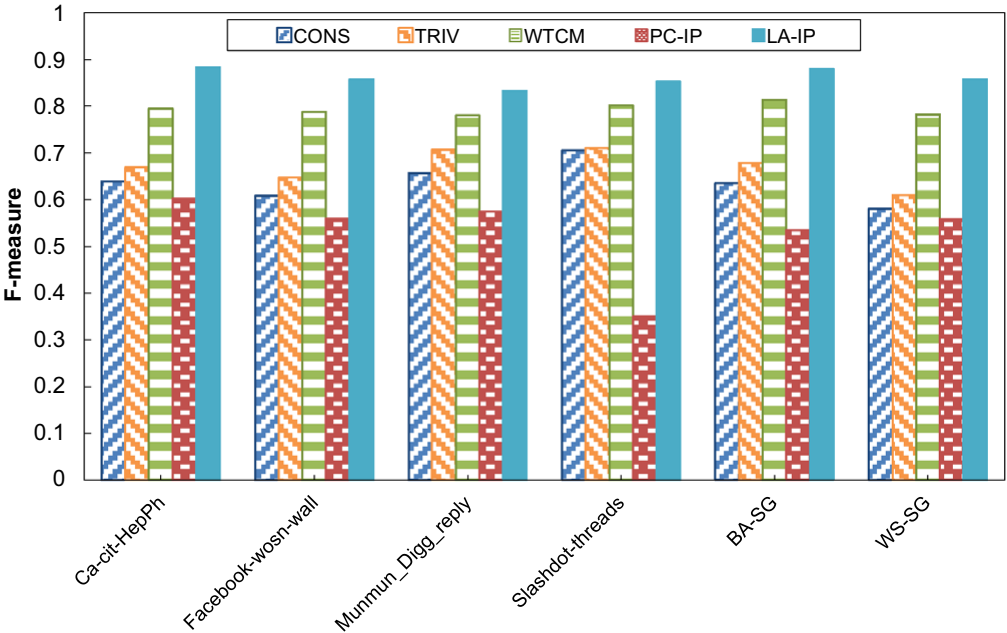
The results of the algorithms for prediction of influence probabilities with respect to F-measure.

**Figure 4:**
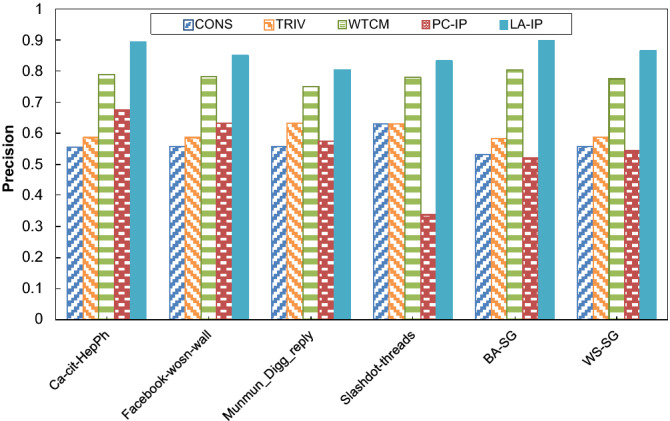
The results of the algorithms for the prediction of influence probabilities with respect to precision.

**Figure 5:**
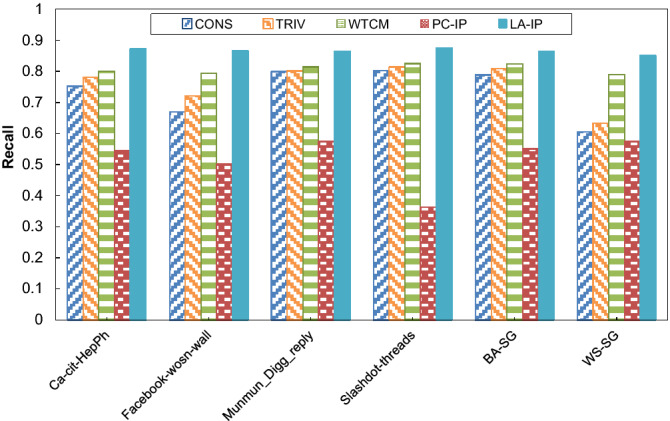
The results of the algorithms for prediction of influence probabilities with respect to recall.

For comparison purposes, we consider some methods as baseline methods under ICM, including (1) a constant influence probability method called CONS, which assigns a constant probability (0.01 in this case) for all links, and (2) a trivalency method called TRIV, which assigns a probability for each link using a uniformly random selection from the probability set {0.001, 0.001, 0.01}, (3) a weighted cascade method called WTCM, which assigns a probability for each link proportional to the inverse in-degree of the incident node and (4) a pure-chance method, which is the same as LA-IP except that the LA residing in each link is replaced by a pure chance automaton. In the pure chance automaton, the actions are selected with equal probabilities^57^. This method is called PC-IP. The experiment results are shown in Fig. 3, Fig. 4 and Fig. 5 for precision, recall, and F-measure, respectively. The results show that the LA-IP algorithm outperforms other methods in terms of recall, precision, and F-measure. In addition, the results show the important role of the LA in guiding the process of learning influence probabilities, indicated by comparing the results with those obtained for the pure-chance automaton version, where learning is absent.

We also performed statistical tests to investigate the significant differences in the results for the prediction based on the estimated influence probabilities. For this purpose, a two-tailed t-test with a confidence level of 95% was executed to compare the algorithms (as listed in Table 2). Based on t-test assumptions, the difference between each pair of algorithms is statistically significant if the difference significance is less than 0.05. Table 2 has three columns: the names of influence maximization algorithms are listed in the first column of the table. The p-value of the t-test corresponding to LA-IP vs. other algorithms is reported in the second column. The third column of Table 2 consists of three signs ‘✓’, ‘✗’ or ‘ ~ ’: if the performance of LA-IP, in terms of influence spread, is significantly better than the compared algorithm, the sign ‘✓’ is denoted in the corresponding cell. The sign ‘✗’ is used when the performance of LA-IP is significantly worse than the compared algorithm. The sign ‘ ~ ’ denotes that LA-IP is similar to the compared algorithm in terms of the influence spread. For example, to compare LA-IP and CONS in terms of the F-measure, the p-value of 3.49e-08 indicates that LA-IP outperforms CONS. From the statistical test results, one can conclude that, in all cases, the results produced by the proposed algorithm (LA-IP) are significantly better than those of other methods with respect to all the test measures (F-measure, precision, and recall). This may mean that the results of LA-IP are more appropriate for estimating influence probabilities. In addition, relatively similar behavior is seen for different types of test networks with respect to precision, recall, and F-measure.

**Table 2:** Results of t-test for comparing the performance of LA-IP versus other algorithms in terms of F-measure.

Algorithms	F-measure		Precision		Recall	
	*p*-Value	Result	*p*-Value	Result	*p*-Value	Result
CONS	3.49e-08	✓	9.48e-09	✓	1.12e-05	✓
TRIV	2.93e-08	✓	8.71e-09	✓	1.58e-05	✓
WTCM	5.73e-07	✓	9.92e-06	✓	8.16e-09	✓
PC-IP	3.64e-08	✓	1.29e-07	✓	1.63e-08	✓

“✓”, “✗”and “~ ” indicate that the performance of the LA-IP is better than, worse than, or similar to that of the compared algorithm, respectively, at a 0.05 level of significance by t-test.

##### Experiment II

This experiment was performed to investigate the convergence behavior of LA-IP in terms of the *Geweke Markov Chain Monte Carlo* (MCMC) convergence diagnostic. The *Geweke* MCMC convergence diagnostic tracks the convergence without ground truth in MCMC sampling applications. The Geweke diagnostic determines the convergence of a single Markov chain, and it can detect when a sample is sufficient for use; hence the sampling algorithm can be stopped. The *Geweke* diagnostic computes the z-statistic of a single sequence of samples as follows

9 $$z = \frac{{E\left[ {X_{i} } \right] - E\left[ {X_{j} } \right]}}{{\left( {Var\left( {X_{i} } \right) + Var\left( {X_{j} } \right)} \right)^{\frac{1}{2}} }}$$

where ${X}_{i}$ is the set of beginning samples from a sequence of samples and ${X}_{j}$ is the set of end samples from a sequence of samples. As the number of samples increases, the sets ${X}_{i}$ and ${X}_{j}$ diverge, and the correlation between them is limited. Hence, based on the law of large numbers, the value of $z$ becomes normally distributed with mean 0 and variance 1. When all values of $z$ fall in the interval [− 1, 1], the sample is sufficient, and the sampling algorithm can be stopped.

**Figure 6:**
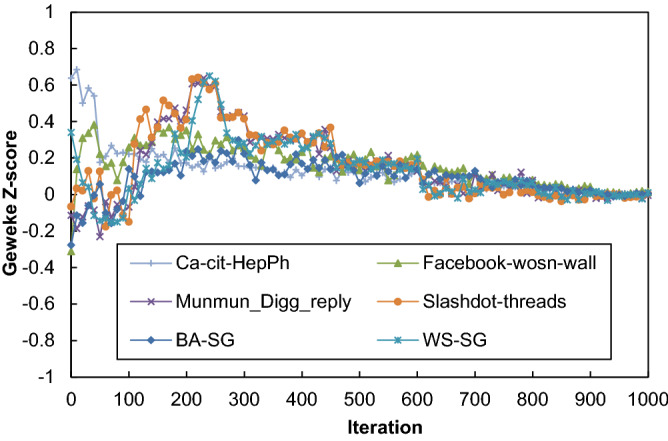
The plot of the *Geweke* Z-score versus iteration number for LA-IP on the stochastic test networks.

For this experiment, ${X}_{i}$ and ${X}_{j}$ are chosen from the first 10% of the beginning samples and the last 50% of the end samples, respectively, from the sequence of samples for each link. We plot the average value of $z$ for each test network for the learning influence probabilities versus iteration number plots. In these plots, the iteration number is the timestamp for each network. The results of this experiment for the stochastic test networks are presented in Fig. 6. From the results, one can observe that (1) at the beginning of the iterations, the values of $z$ have drastically oscillated. This may be because the algorithm varied among the actions to take samples. (2) when the iterations are increased, the oscillation gradually declined in late iterations, the reason behind may that may reaching near sufficient samples. (3) for the whole iteration all the values of $z$ fall in the interval [−1, 1] for all the runs. This means that the proposed algorithms for learning influence probabilities using the initial random samples gradually learn the proper influence probabilities.

## Conclusion

In this paper, we discussed that a stochastic graph is an appropriate model for social networks due to the dynamic, unpredictable, uncertain, and time-varying nature of OSNs. We then designed a diffusion model for stochastic graphs. An LA-based algorithm was developed to estimate influence probabilities using user activities under the situation that the PDF of the weights associated with the links of the stochastic graph is unknown. The effectiveness of the proposed diffusion model and the proposed algorithms was tested using real and artificial stochastic networks.

## Supplementary Information

Supplementary Information.

## Data Availability

All data generated or analyzed during this study are included in this or its supplementary information files. The data that support the findings of this study are available in two parts: real and artificial data. The real data are publicly available at THE KONECT PROJECT NETWORKS repository: http://konect.cc/networks, consisting ca-cit-HepPh: at http://konect.cc/files/download.tsv.ca-cit-HepPh.tar.bz2, Facebook-wosn-wall at: http://konect.cc/files/download.tsv.facebook-wosn-wall.tar.bz2, Munmun_Digg_reply at: http://konect.cc/files/download.tsv.munmun_digg_reply.tar.bz2, and Slashdot-threads at http://konect.cc/files/download.tsv.slashdot-threads.tar.bz2. The artificial data that support the findings of this study are available from the corresponding author, A. R., upon request.

## Code availability

The codes can be obtained from the corresponding author, A. R. at reasonable request.
